# Self-assembled single-crystal bimodal porous GaN exhibiting a petal effect: application as a sensing platform and substrate for optical devices†

**DOI:** 10.1039/d2na00164k

**Published:** 2022-06-27

**Authors:** Taishi Kimura, Masakazu Murase, Yuri Yamada, Norihiro Mizoshita, Daisuke Nakamura

**Affiliations:** Toyota Central R&D Labs., Inc. Nagakute Aichi 480-1192 Japan taishi@mosk.tytlabs.co.jp

## Abstract

This paper investigates the petal effect (hydrophobicity and strong adhesion) observed on single-crystal bimodal porous GaN (porous GaN), which has almost the same electrical properties as bulk GaN. The water contact angles of porous GaN were 100°–135° despite the intrinsic hydrophilic nature of GaN. Moreover, it was demonstrated that the petal effect of porous GaN leads to the uniform attachment of water solutions, enabling highly uniform and aggregation-free attachment of chemicals and quantum dots. These results indicate that porous GaN can be applied in quantum dot light-emitting diodes and as an analytical substrate.

## Abstract 2

Petal effect (hydrophobicity and strong adhesion) exhibited by single-crystalline bimodal-porous-GaN creating high uniform attachment of chemical molecules and quantum dots.

## Graphical abstract

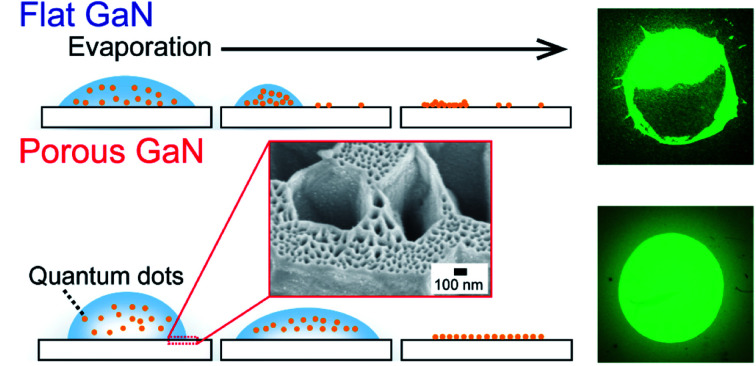

## 1. Introduction

The implementation of bio-inspired designs is one of the most successful methods for promoting technological innovations. For example, water repellency observed on the surfaces of lotus leaves has been studied for over 20 years.^1–5^ Based on this feature of lotus leaves, the term “lotus effect” has been coined, which refers to the low adhesion of water droplets on superhydrophobic surfaces; this effect can be exploited for the development of self-cleaning coatings and textiles.^1–5^ In addition, considerable research conducted in recent years has focused on the petal effect, which is the high adhesion of water droplets on superhydrophobic surfaces, based on the features of rose petals.^6–9^ The petal effect has attracted widespread interest in various fields, such as for droplet trapping in dry environments,^10^ water transportation and harvesting,^11,12^ oil/water separation,^13^ and localized chemical or biological reactions.^14^ Functional materials exhibiting unique wetting properties such as the petal effect have great potential for use as substrates in molecule pinning and biological or chemical analysis as well as in sensing and detection.^9,11^

Gallium nitride (GaN) is a promising functional material for use in various electronic and photonic applications because it has many exceptional properties, such as easily modifiable electrical conductivity (both n-type and p-type), carrier concentration controllability, high carrier mobility, bandgap tunability, non-toxicity, and high thermal/chemical stability.^15–19^ Various electronic devices based on GaN, such as optoelectronic, high-frequency electronic, and power electronic devices, have been studied and commercialized.^18,19^ The unique wetting properties of GaN have generated considerable recent research interest such as applications in sensorics,^20,21^ catalyst supports in photoelectrodes,^22^ and inkjet printing for aggregation-free quantum dot (QD) micro-light-emitting diodes (micro-LEDs).^23^ However, the cost-effective functional fabrication of GaN that allows contact angle control and that exhibits the lotus effect and the petal effect remains difficult.^24^ To the best of our knowledge, the realization of the petal effect on GaN surfaces has never been reported previously.

**Fig. 1:**
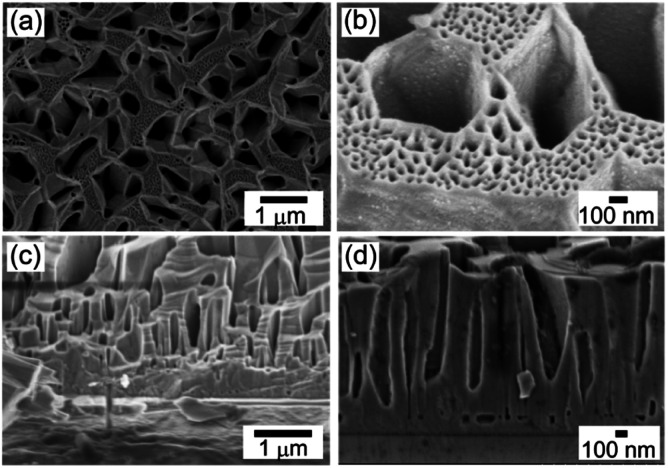
Scanning electron microscopy (SEM) images of porous GaN. (a and b) Plane view images of porous GaN. (c and d) Cross-sectional images of porous GaN at the growth interface.

This article reports the unique wetting behavior of bimodal porous GaN, which possesses almost the same electrical properties as those of bulk GaN. In a previous study, we demonstrated that the fabrication of single-crystal bimodal (meso/macro) porous GaN (porous GaN) (as shown in Fig. 1) and its applicability in photochemistry, electrochemistry, and catalysis.^22^ The significance of the present work lies in the demonstration that porous GaN exhibits the petal effect (hydrophobicity and strong adhesion) despite the intrinsic hydrophilic nature of GaN. Furthermore, it reveals that the petal effect of porous GaN leads to the uniform attachment of water solution, resulting in highly uniform and aggregation-free attachment of chemicals and QDs.

## 2. Experimental

### 2.1 Preparation of flat GaN

The flat GaN samples were commercially available 2.0 μm-thick *c*-face (0001) GaN layers grown on sapphire substrates (MO templates) *via* metal–organic chemical vapor deposition (MOCVD). The flat GaN : Si samples, as shown in Fig. 5(a) and (b), were fabricated *via* halogen-free vapor phase epitaxy (HF-VPE). Details regarding the growth configuration and conditions have been reported previously.^25^

### 2.2 Preparation of porous GaN

The porous GaN samples were fabricated through HF-VPE^22,25–30^ on MOCVD GaN on sapphire substrate (MO template) (ESI†).^22,25–30^ The bimodal meso/macro porous structure was a self-assembled structure generated by the anti-surfactant effect of B during GaN growth.^22,26^ Details regarding the growth configuration and conditions have been reported elsewhere.^22,26,28^ The surface morphology and crystal quality of porous GaN were assessed by SEM and X-ray diffraction analysis, whereas the identification and concentration determination of B, C, O, and Si impurities were performed by SIMS.

### 2.3 Preparation of flat GaN-F9 (ref. 31)

Flat GaN-F9 was prepared by exposing flat GaN to trimethoxy(1*H*,1*H*,2*H*,2*H*-nonafluorohexyl)silane (FAS-9) vapor at 150 °C for 1 h in an Ar atmosphere. Flat GaN-F9 was sonicated in acetone to remove excess FAS-9 and then dried on a plate heated at 50 °C.

### 2.4 Contact angle measurements

Contact angle measurements were carried out using a DMo-501 manufactured by Kyowa Interface Science Co., Ltd. The static contact angles (*θ*_S_) values of the water droplets (0.5 μL) were calculated as the averages of the contact angles at five different points. The dynamic contact angle (*θ*_D_) values were measured every 5 s after the water droplets (1.0 μL) contacted the sample surfaces. The measurements were conducted until the water droplets disappeared from the surface by evaporation. The advancing and receding contact angles (*θ*_A_ and *θ*_R_, respectively) were measured by expanding and contracting the volume of the water droplet on the sample surface. *θ*_A_ was measured as the expansion of the water droplet volume from 0.5 to 5.5 μL, and *θ*_R_ as the contraction of the water droplet volume from 5.5 μL to 0 μL. These measurements were also conducted at room temperature in air.

### 2.5 LDI time of flight mass measurements

Laser desorption/ionization (LDI) time-of-flight (TOF) mass spectroscopy conducted using an autoflex® maX system manufactured by Bruker. An aqueous solution containing DHB (10 mM) and trifluoroacetic acid (0.1 vol%) was spotted on the sample surface and dried at room temperature. LDI TOF mass spectra were measured at 100 μm intervals in the analytical area in which the sample solution was spotted. The measurement was conducted in the reflectron mode, and the laser power, detector gain, frequency, laser diameter, and number of laser shots per one spot were set to 50%, 1600 V, 100 Hz, 100 μm, and 100 shots, respectively. The images that visualized the signal intensity distribution of 2,5-dihydroxybenzoic acid (DHB) (Fig. 4(a)–(c)) were drawn based on the LDI TOF mass spectra using the flexImaging software.

### 2.6 QDs

CdSe/ZnS core–shell-type QDs (fluorescence wavelength: 540 nm, 1 mg mL^−1^) in water (Type 918814 Sigma-Aldrich) were used.

## 3. Results

### 3.1 Electrical properties of porous GaN

Porous GaN with many porous structures shown in Fig. 1 was grown on MO template as a single crystal, as reported previously (ESI†).^22^ The large pores are 300–1500 nm in diameter and 0.3–1.5 μm in depth. The mesoscale pores with a diameter of 20–100 nm are formed on the top surface of porous GaN. The single-crystal nature and high-quality crystallinity of porous GaN were confirmed by X-ray diffraction measurements (Fig. S1, ESI†). The highly ordered framework is expected to result in high electrical and thermal conductivities and good homogeneity over a large area, which are useful for various applications such as electronic devices, catalyst supports and inkjet printing of QDs for micro-LEDs. Thus, we analyzed the electrical properties of porous GaN.

**Fig. 2:**
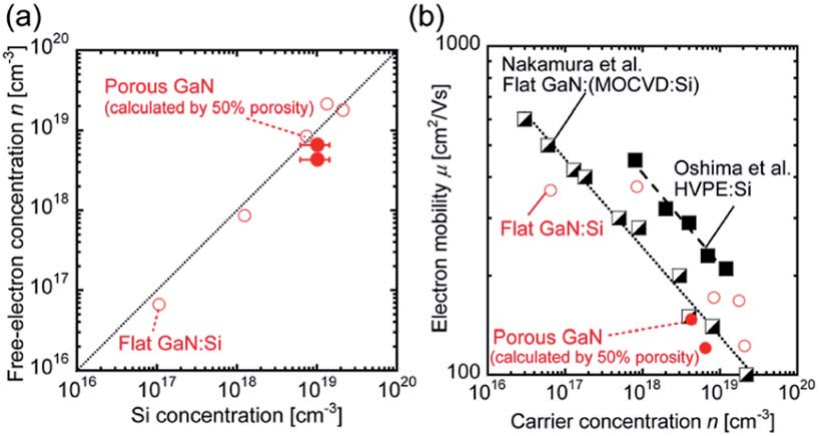
Comparison of the electrical properties of porous and flat GaN. (a) Relationship between the Si concentration and net donor concentration for porous and flat GaN grown by HF-VPE. (b) Dependence of electron mobility on carrier concentration for GaN layers at 300 K.

Secondary ion mass spectrometry (SIMS) was performed to identify the concentrations of the donor dopant impurity Si (Fig. S2, ESI†). The donor dopant impurity of the Si concentration was approximately 1 × 10^19^ atoms per cm^3^. van der Pauw–Hall measurements were performed to investigate the net donor concentration, specific resistance, and electron mobility at 300 K. Fig. 2(a) shows the relationship between the Si concentration and free-electron concentration *n* for porous GaN and the previously reported flat GaN grown by HF-VPE.^25^ The porous GaN data presented in Fig. 2(a) and (b) were calculated assuming that porous GaN has 50% porosity. The assumed 50% porosity was roughly determined in reference to the pore coverage ratio estimated from the binarized SEM image (Fig. S3, ESI†). The free-electron concentrations of porous GaN are almost equal to the Si concentrations as shown in Fig. 2(a). Thus, the free-electron concentration of porous GaN can be controlled by adjusting the Si doping concentration, as in the case of flat GaN.

Fig. 2(b) illustrates the dependence of the electron mobility on the carrier concentration for the GaN layers at 300 K. The electron mobility data for MOCVD-GaN on a sapphire substrate and hydride vapor phase epitaxy (HVPE)-GaN on freestanding GaN substrates are also shown for comparison.^32,33^ Although the electron mobility of porous GaN is lower than that of freestanding GaN, which has a low threading dislocation density, the values are almost the same as those of MOCVD-GaN, which has a dislocation density similar to that of porous GaN. Surprisingly, the electrical properties of porous GaN with a bimodal porous structure are almost identical to those of single-crystal flat GaN (Fig. S4, ESI†). These results indicate that the electrical properties such as the carrier concentration and carrier mobility of porous GaN are controllable, as in the case of flat GaN.

### 3.2 Wetting properties of porous GaN and flat GaN

**Fig. 3:**
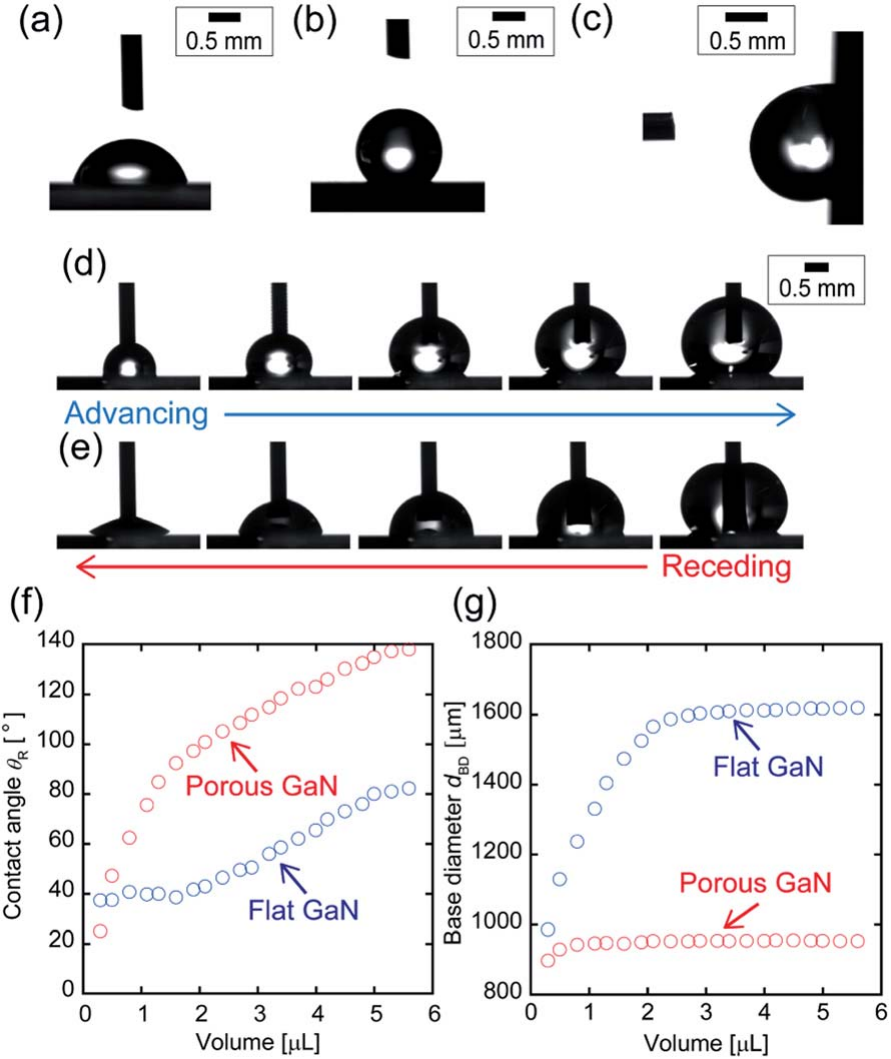
Optical micrographs of water droplets on (a) flat GaN, (b) porous GaN, and (c) porous GaN at a 90° tilt angle. (d and e) Optical micrographs of a water droplet on porous GaN when the water droplet is inflated and deflated, respectively. (f and g) Receding contact angle and base diameter of flat and porous GaN *versus* the volume of the water droplet, respectively.

Fig. 3(a) and (b) show the micrographs of the water droplets on flat and porous GaN surfaces, respectively. Flat GaN exhibits hydrophilicity and possesses a contact angle of 76° ± 1.6°, which is almost the same as that reported for GaN films.^34^ Conversely, porous GaN exhibits hydrophobicity and possesses a contact angle of 133° ± 1.4°.

Fig. 3(c) shows the micrograph of a water droplet on porous GaN at a 90° tilt angle. Although porous GaN exhibits hydrophobicity, the droplet remains stuck under the 90° tilted condition. This feature reveals that the water droplet strongly adheres to porous GaN. The droplet on porous GaN did not roll off even if porous GaN was turned upside-down (not shown). Fig. 3(d) and (e) present the micrographs of inflated and deflated droplets on porous GaN during the advancing and receding cycles, respectively, and Fig. 3(f) and (g) show the receding contact angle *θ*_R_ and base diameter *d*_BD_ plotted against the water volumes of flat and porous GaN. The advancing angle of porous GaN can be as high as 138°, and the receding angle continues to decrease to ∼20° as the volume is reduced. Moreover, the contact angle hysteresis of porous GaN exceeds 110° because of the pinned contact line corresponding to a constant base diameter during receding. Thus, the wetting properties of porous GaN demonstrate its hydrophobicity and strong adhesion to water droplets; therefore, porous GaN exhibits the so-called petal effect.^6^

### 3.3 Pore coverage ratio on the porous GaN and wetting properties of flat GaN, flat GaN-F9, and porous GaN

**Fig. 4:**
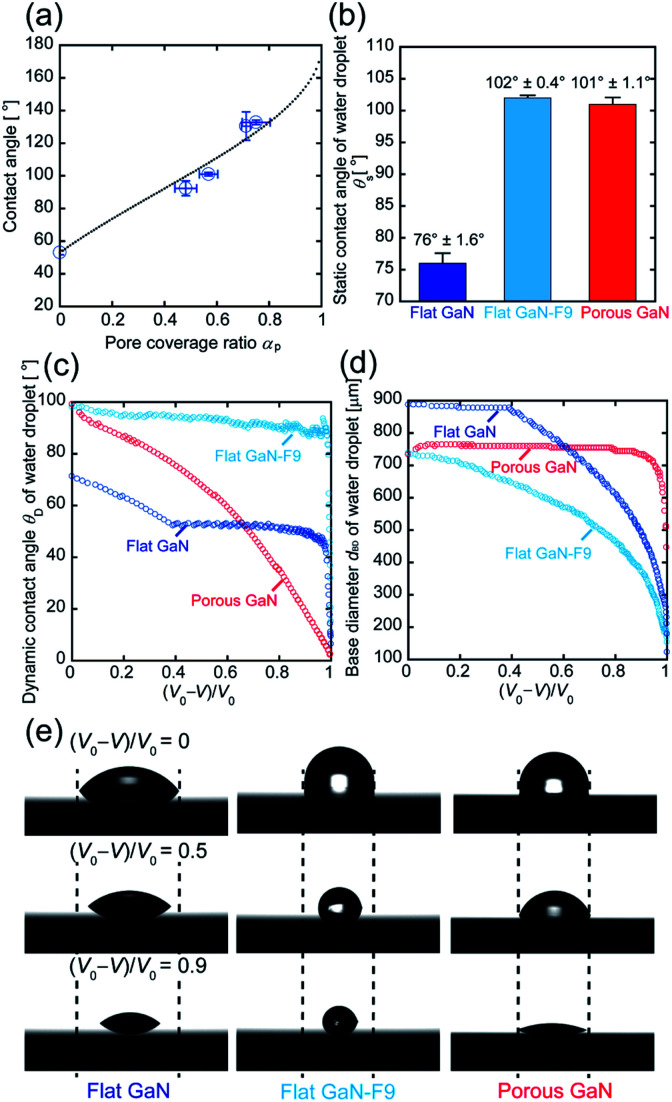
(a) Relationship between *α*_p_ of porous GaN and contact angle. (b) *θ*_S_ of water droplets (0.5 μL) on the surfaces of flat GaN, flat GaN-F9, and porous GaN. (c and d) *θ*_D_ and *d*_BD_ of the water droplets on flat GaN, flat GaN-F9, and porous GaN during the vaporization process. (e) Images of the water droplets on flat GaN, flat GaN-F9, and porous GaN during the vaporization process ((*V*_0_ − *V*)/*V*_0_ = 0, 0.5, 0.9). Here, the initial and remaining volumes of the droplet are defined as *V*_0_, and *V*, respectively.

To understand the mechanism of the increase in the contact angle of a water droplet on porous GaN in relation to that on flat GaN, we investigated the relationship between the pore (meso/macro pore) coverage ratio and contact angle of porous GaN. Fig. 4(a) depicts the relationship between the pore coverage ratio (*α*_p_) of porous GaN and the contact angle. Here, *α*_p_ of porous GaN was obtained from the SEM images of each porous GaN surface, which were converted to the binary form *via* image processing (Fig. S3, ESI†).

To demonstrate the difference between porous GaN having a petal effect and flat GaN with simple hydrophobicity, we prepared porous GaN with water contact angles similar to those of water-repellent flat GaN. Water-repellent flat GaN was prepared by surface coating with FAS-9. The static contact angles (*θ*_S_) of flat GaN, flat GaN coated with FAS-9 (flat GaN-F9), and porous GaN are shown in Fig. 4(b). The *θ*_S_ values of flat GaN-F9 and porous GaN are approximately 100°.

Fig. 4(c) presents the dynamic contact angle (*θ*_D_) during the vaporization process of the water droplets on flat GaN, flat GaN-F9, and porous GaN. An almost constant *θ*_D_ value is evident for flat GaN and flat GaN-F9 during water droplet volume reduction. Conversely, the *θ*_D_ value of porous GaN constantly decreases during water droplet volume reduction. Fig. 4(d) shows the base diameter *d*_BD_ during the vaporization of flat GaN, flat GaN-F9, and porous GaN. As shown in Fig. 4(d), the *d*_BD_ values of flat GaN and flat GaN-F9 decrease as the volume of the water droplet decreases. In contrast, the *d*_BD_ value of porous GaN remains constant, which could be the edge of the water droplet is pinned onto porous GaN, as shown in Fig. 4(e). These results for porous GaN agree well with those in Fig. 3. Thus, porous GaN exhibits the petal effect. These results also demonstrate that porous GaN has a different vaporization process than flat GaN and merely hydrophobic flat GaN-F9.

### 3.4 Uniform attachment of molecules or nanomaterials on the porous GaN

The unique wetting properties of porous GaN may facilitate the uniform attachment of molecules or nanomaterials when an aqueous solution or aqueous dispersion is employed; therefore, experiments of coating a low-molecular-weight compound and QD were performed.

**Fig. 5:**
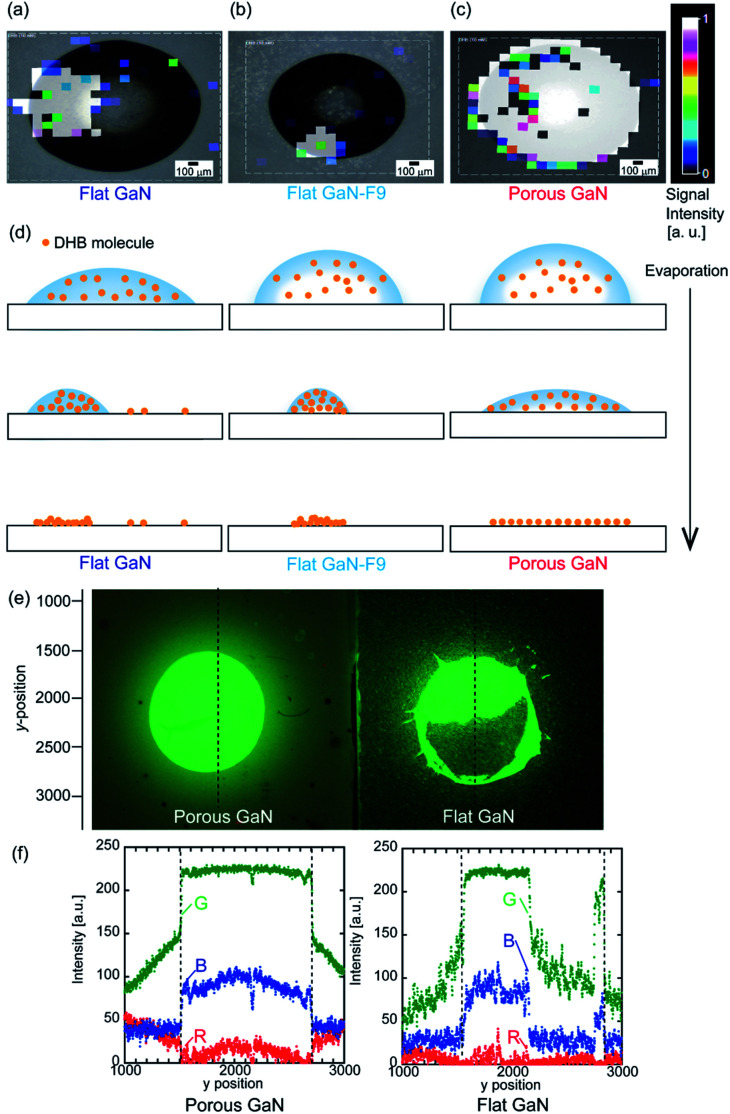
Mapping distributions of LDI signal intensities of DHB (10 mM) on the surfaces of (a) flat GaN, (b) flat GaN-F9, and (c) porous GaN. Each water droplet image before LDI measurement is displayed for superposition on the signal intensity distributions. (d) Schematics of the evaporation processes of water droplets containing DHB molecules for each substrate. (e) Photograph comparing the attached areas of CdSe/ZnS core–shell-type QDs (fluorescence wavelength: 540 nm) on porous and flat GaN under ultraviolet light. The drop volume was 5 μL. (f) RGB line scan data of porous and flat GaN obtained along the black dashed lines in (e). The black dashed lines in (f) represent the *y* positions at which liquid droplets QDs were deposited.

Uniform wetting properties due to the petal effect of porous GaN toward a low-molecular-weight compound were examined. A water solution of 2,5-dihydroxybenzoic acid (DHB, 10 mM) was applied to the flat GaN, flat GaN-F9, and porous GaN substrates and dried at room temperature. To identify the attachment region of DHB molecule on the each GaN, the LDI mass spectrometry measurement was performed. Fig. 5(a)–(c) depict the signal intensity distributions of DHB (10 mM) for the surfaces of flat GaN, flat GaN-F9, and porous GaN upon LDI measurement, respectively. Each liquid solution droplet of DHB (10 mM) on the substrate before LDI measurement is displayed for superposition on the signal intensity distributions in Fig. 5(a)–(c) (Fig. S5, ESI†). The drop volume of the DHB liquid solution is the same on each substrate. Fig. 5(a)–(c) show that the signal intensity distributions of porous GaN exhibit drastically different profiles in relation to those of flat GaN and flat GaN-F9. The mapping distribution profiles of flat GaN and flat GaN-F9 do not match the droplet image of the liquid solution before LDI measurement, as shown in Fig. 5(a) and (b). Thus, partial aggregation of DHB molecules occurs during the evaporation of the liquid solution (Fig. 5(a), (b) and (d)). Conversely, the profile of porous GaN perfectly matches the droplet image of the liquid solution before measurement, and porous GaN has a very uniform signal intensity (Fig. 5(c)). The mapping distribution profile of porous GaN is attributed to the unique vaporization process of the liquid solution droplet on the porous GaN substrate (Fig. 5(d)).

Porous structures have been widely utilized for surface-assisted LDI mass spectrometry because the porous structure can increase the absorption efficiency of light and promote analyte desorption/ionization.^35^ Nie *et al.* reported that porous GaN with Ag nanoparticles as cationization agents can be applied for LDI mass spectroscopy (LDI-MS).^17^ Therefore, porous GaN may be utilized as an analytical substrate in applications such as LDI-MS.

Then, to demonstrate the wide usability of porous GaN owing to its unique wetting properties caused by the petal effect, the wetting properties of porous GaN toward QDs were studied. CdSe/ZnS core–shell-type QDs (fluorescence wavelength: 540 nm) were used for this experiment. An aqueous dispersion of QDs (5 μL) was applied to the porous and flat GaN substrates, which were dried at room temperature. Fig. 5(e) compares the attached areas of CdSe/ZnS core–shell-type QDs on porous and flat GaN under ultraviolet light. It is obvious that the attachment of QDs on porous GaN is remarkably more uniform than that on flat GaN. The image in Fig. 5(e) was analyzed; Fig. 5(f) presents the red-green-blue (RGB) color intensities of line scan data of porous and flat GaN obtained along the black dashed line in Fig. 5(e). The image analysis was performed using the MATLAB software (Fig. S6, ESI†). Fig. 5(f) clearly reveals that the QD emission intensities for porous GaN are more uniform than those for flat GaN on deposited region of liquid droplets QDs.

## 4. Discussion

As shown in the Results section, the electrical properties, the crystallinity, and the crystal orientation are almost identical in porous GaN and flat GaN. Thus, the petal effect (hydrophobicity and strong adhesion) of porous GaN should be caused by the surface morphology on porous GaN as shown in Fig. 1. Two wetting models, namely, the Cassie–Baxter and Wenzel states of water droplets against rough surfaces are well known.^36,37^ Assuming that the wetting state of porous GaN is the Wenzel state type, the contact angle of porous GaN should decrease as the *α*_p_ value of porous GaN increases because the contact angle of flat GaN is <90°. However, as the *α*_p_ value of porous GaN increases, the contact angle of porous GaN increases, as shown in Fig. 4(a). In contrast, assuming that the wetting state of porous GaN is the Cassie–Baxter state, as the *α*_p_ value of porous GaN increases, the contact angle of porous GaN should increase. The apparent contact angle (*θ*_CB_) on the rough surface of the Cassie–Baxter equation can be written as follows:^36,37^

1 cos *θ*_CB_ = *f*_1_ cos *θ*_F_ + *f*_1_ − 1,

where *f*_1_ is the fraction of the GaN surface area wetted by water, and *θ*_F_ is the contact angle of the droplet on flat GaN. We assumed *f*_1_ and *θ*_F_ to be equal to 1 − *α*_p_ and 76° ± 1.6°, respectively. The black dotted line in Fig. 4(a) was estimated by using the Cassie–Baxter equation. The contact angle results of porous GaN agree well with the values obtained using the Cassie–Baxter equation. This finding indicates that the high contact angles of porous GaN originate from the meso/macro pores on the surface and that the contact angle can be controlled by *α*_p_, which depends on the porous GaN growth conditions such as doped B concentration and growth rate.^22^

Spencer *et al.* reported the relationships between several rough structures at the nanometer scale and their wetting states.^38^ Their study revealed that a hydrophobic surface state can be formed by using hydrophilic materials in the maintainable, metastable Cassie–Baxter state.^38^ Flat GaN is hydrophilic; therefore, it is very probable that the hydrophobicity of porous GaN is attributable to the presence of the metastable Cassie–Baxter state; the porous rough structure of porous GaN should also be mainly filled with air.

There are two possible mechanisms that could produce the unique wetting properties of water droplets on porous GaN. One is an energy barrier that exists as water fills the grooves.^38,39^ When water fills the grooves, whose cross-sections become larger toward the bottom (the so-called undercut structure), the liquid–air interface must expand, increasing the equilibrium free energy.^38,39^ Assuming that porous GaN has an undercut structure, an energy barrier should exist during the water-filling process because the liquid–vapor interface must be expanded for water penetration in the undercut structure.

The other possible mechanism is a negative pressure of the air trapped in the closed tube, as reported by Jiang *et al.*^40,41^ As shown in Fig. 1(c) and (d), the bottoms of the macropores in porous GaN appear to have a closed tube structure. The adhesive force of porous GaN for the water droplets may originate from negative pressure produced by the different volumes of the sealed air in the closed tubes.^40,41^ The adhesive force of porous GaN for the water droplets could be explained by the macropore structure of porous GaN; therefore, the meso/macropore structures of porous GaN confer unique wetting properties to porous GaN.

Our findings add to the existing body of knowledge on porous GaN.^22^ These results demonstrate the usability of porous GaN as analytical substrates such as those utilized in LDI-MS, as catalyst support substrates for photochemical or electrochemical reactions, and in inkjet printing of QDs for micro-LEDs.

## 5. Conclusions

We demonstrated that single-crystal porous GaN with a hybrid meso/macroporous structure exhibits the petal effect. The petal effect of porous GaN imparts considerably different properties in relation to those of flat GaN, such as excellent uniformity of QD attachment and no aggregation. Moreover, the free-electron concentration can be controlled by adjusting the doping concentrations of porous GaN, and the electron mobility of porous GaN is almost the same as that of flat GaN. These findings could enable the application of porous GaN in QD-LEDs, as catalyst supports, and as analytical substrates for LDI-MS.

## Author contributions

T. K. conducted all growth trials and most of the characterizations. M. M. and Y. Y. carried out the contact angle and LDI measurements and interpreted the results. N. M. and D. N. initiated and guided the study. T. K. wrote the manuscript and ESI.† All authors contributed to the discussion.

## Conflicts of interest

There are no conflicts to declare.

## Supplementary Material

NA-004-D2NA00164K-s001
