# Supplementary material for: Self-assembled single-crystal bimodal porous GaN exhibiting a petal effect: application as a sensing platform and substrate for optical devices
Source: Nanoscale Adv. 2022 Jun 27;4(18):3718–24. doi: 10.1039/d2na00164k (PMC9470053; doi:10.1039/d2na00164k)
Supplement: NA-004-D2NA00164K-s001 [file NA-004-D2NA00164K-s001.pdf]

Electronic Supplementary Material (ESI) for Nanoscale Advances.
This journal is © The Royal Society of Chemistry 2022

Supplementary Information

Self-assembled single-crystal bimodal porous GaN exhibiting a petal effect: Application as a sensing platform and substrate for optical devices

Taishi Kimura*, Masakazu Murase, Yuri Yamada, Norihiro Mizoshita, and Daisuke Nakamura

Toyota Central R&D Labs., Inc., Nagakute, Aichi 480-1192, Japan

*Corresponding author: taishi@mosk.tytlabs.co.jp

Table of Contents

1. Growth of porous GaN by halogen-free vapor-phase epitaxy ------------------------ S-3

2. SEM analysis -- S-3

3. X-ray rocking curve --- S-4

4. Secondary ion mass spectrometry -- S-6

5. Estimation of the pore coverage ratio α_p of porous GaN ------------------------------ S-8

6. van der Pauw-Hall measurements and electrical properties of porous GaN -------- S-10

7. Mass spectra by LDI measurements -- S-13

8. Quantum dots photographs and image analysis of those photographs --------------- S-14

9. Photoluminescence spectra of quantum dots on porous GaN and on flat GaN----- S-16

10. References --S-18

1. Growth of porous GaN by halogen-free vapor-phase epitaxy

Bimodal porous single-crystal GaN (porous GaN) were fabricated by halogen-free vapor phase epitaxy (HF-VPE)[1-6] using graphite crucibles with a pyrolytic boron nitride coating. A 2 μm-thick GaN template grown via metalorganic chemical vapor deposition on a sapphire substrate was used as the seed crystal (MO template). The temperature of the growth susceptor was 1353 K, and the crucible was heated to approximately 1410–1480 K by radio frequency irradiation. The growth pressure was 4 kPa and growth duration was 9 min.

2. SEM analysis

Field-emission scanning electron microscopy (FE-SEM) images were acquired using a Hitachi S-4800 instrument. The accelerating voltage was approximately 3 kV and the emission current was approximately 10 μA.

3. X-ray rocking curves

Figure S1 (a, b) shows the normalized rocking curves for the (0002) and ($11\bar{2}2$) reflections of porous GaN, respectively. The full width at half maximum (FWHM) values in the ω-scan XRC data for the porous GaN layer are 292 and 699 arcsec for the (0002) and ($11\bar{2}2$) reflections, respectively. These FWHM values are slightly smaller than those obtained for the MO-template seed layer (approximately 320 and 760 arcsec, respectively). These results suggested that the crystallinity of porous GaN was slightly higher than that of the MO template (i.e., the seed crystal), and crystal quality degradation such as upon dislocation increase did not occur during porous GaN growth. The ϕ-scan data for the GaN ($11\bar{2}2$) planes in porous GaN from −180 °to 180° are shown in Figure S1 (c). The data show only six peaks due to the equivalent ($11\bar{2}2$) planes. Thus, the porous GaN layer was grown in a homoepitaxial manner on the MO template substrate.

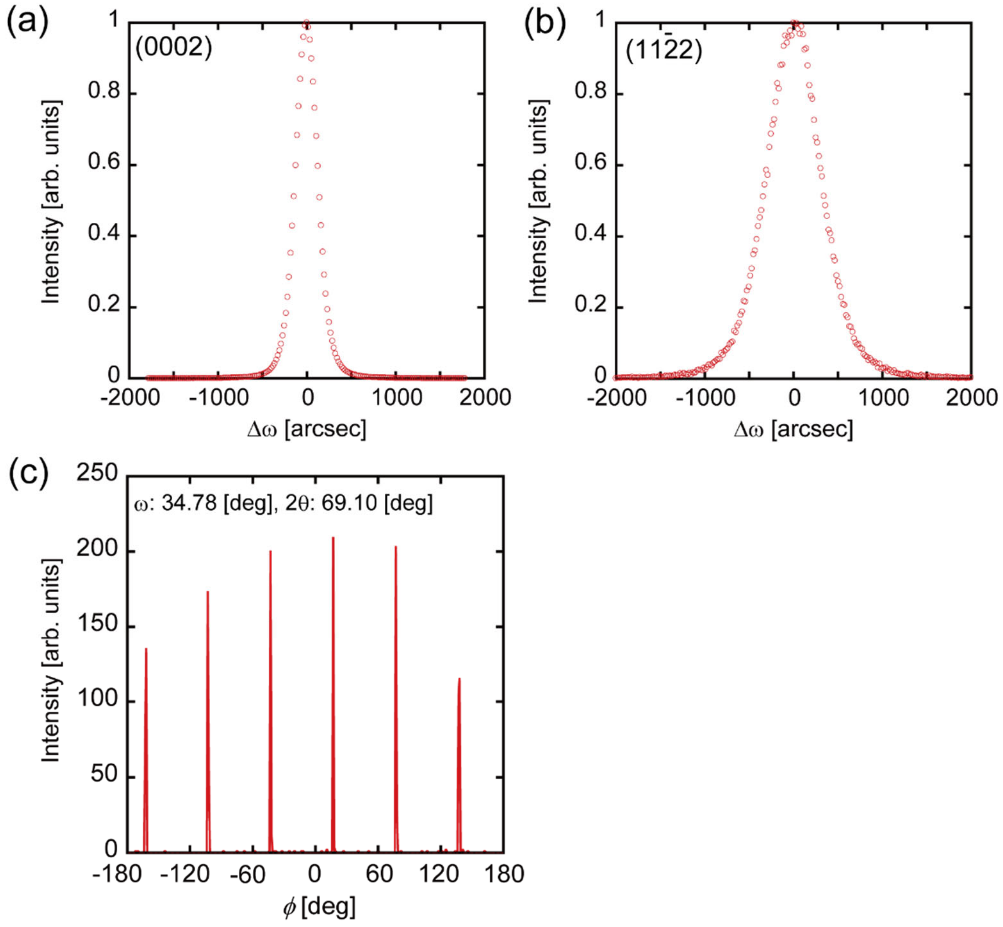

Figure S1. (a, b) Normalized X-ray rocking curves for the (0002) and (11$\bar{2}$2) planes of porous GaN. (c) ϕ-scan XRC data for the (11$\bar{2}$2) planes of porous GaN.

4. Secondary ion mass spectrometry

Secondary ion mass spectrometry (SIMS) analyses were performed to identify the concentrations of anti-surfactant impurity boron for preparing bimodal porous structures, the donor dopant impurity silicon and oxygen, and the compensated impurity of carbon in porous GaN (Figure S2). The resulting data show that porous GaN had a boron concentration of 2×10^{19} atoms/cm^3, which is almost the same as the previously reported value.[7] Considerably high (10^{20}–10^{21} atoms/cm^3) carbon and oxygen concentrations were confirmed, and the depth profiles of carbon and oxygen were quite similar. Porous GaN had a significantly high specific surface area and was exposed to the atmosphere; therefore, the surface of porous GaN should be covered with hydrocarbons or atmospheric species. Thus, the high concentrations of carbon and oxygen could be attributed to the surface contamination of porous GaN and not to porous GaN itself. The donor dopant impurity of the silicon concentration was approximately 1×10^{19} atoms/cm^3. SIMS data were obtained with a CAMECA IMS-6f instrument, at an accelerating voltage of 15.0 kV over an analysis area with a diameter of 30 μm and using Cs^+ ions. The accuracy of the measurement was ±40 % (2σ).

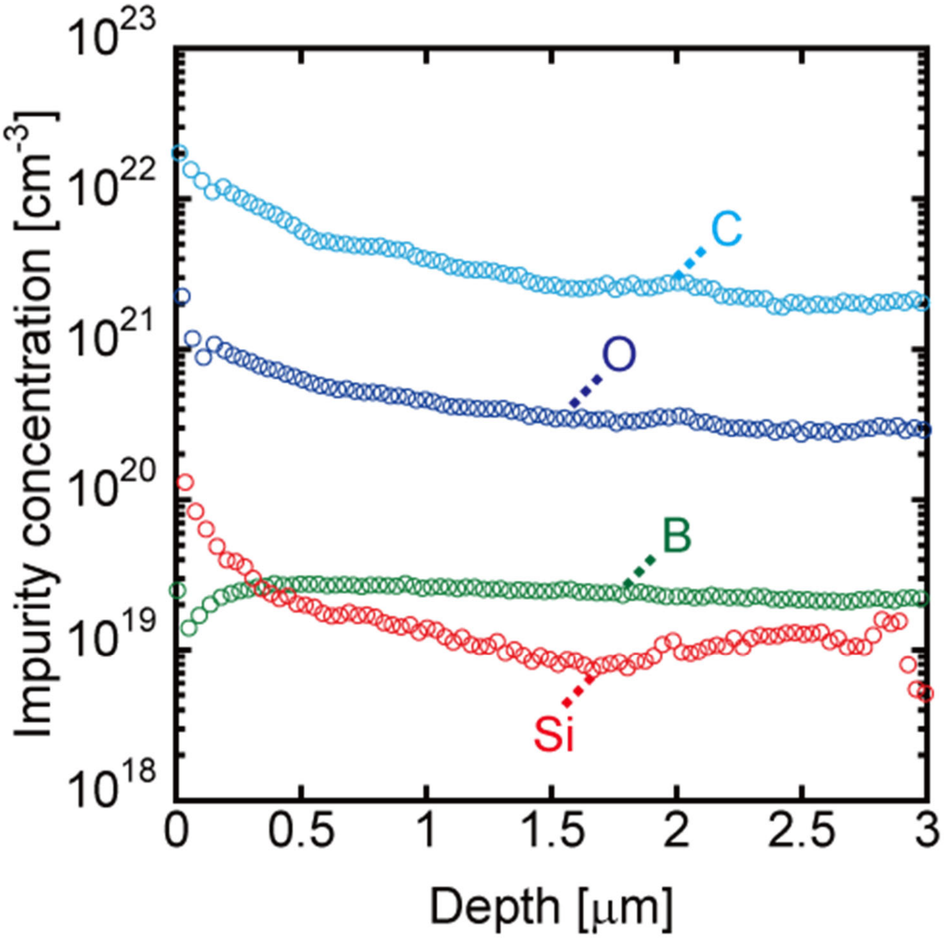

Figure S2. Depth profile of B, C, O, and Si impurities obtained by SIMS.

5. Estimation of the pore coverage ratio α_p of porous GaN

The pore coverage ratio α_p of porous GaN was obtained from the SEM images of each porous GaN surface, which were converted to binary via image processing. Figure S4. represents scanning electron microscope (SEM) images of porous GaN and binarized images of each image. The white colored region on the figure S4(b) and S4(d) show estimated pore region. The estimated pore coverage ratio α_p of figure S4(b) and S4(d) were approximately 71 % and 51 %, respectively. The α_p of porous GaN was calculated by three SEM images of each sample, and the α_p and the error bar of α_p in figure 4(a) of manuscript were the average values and the standard deviation of the α_p, respectively.

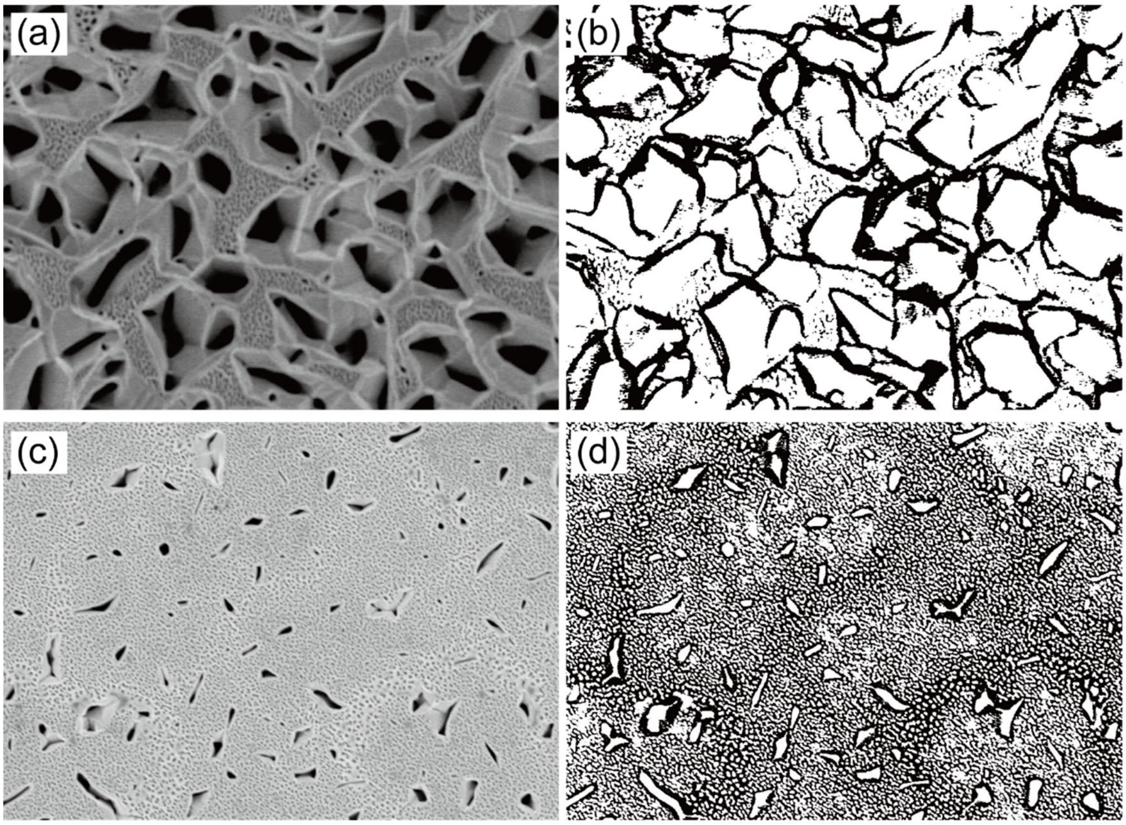

Figure S3. (a, c) Original plan-view SEM images of porous GaN, (b, d) binarized images of SEM images of (a) and (c), respectively.

6. van der Pauw-Hall measurements and electrical properties of porous GaN

To verify the electrical properties of porous GaN, van der Pauw-Hall measurements were conducted at 300 K. A Ti/Al/Ni (20/200/40 nm) ohmic contact was deposited by e-beam evaporation. The measurement apparatus was a RESITEST 8300 (TOYO Corporation).

The free-electron concentration, *N,* was estimated using the sheet carrier concentration, N_{sheet}, obtained from the van der Pauw-Hall measurements.

$$N = \frac{N_{sheet}}{t} \qquad (1)$$

where *t* is the thickness of the porous GaN layer. The corrected free-carrier concentration, N_{correct}, was calculated as follows:

$$N_{correct} = \frac{N_{sheet}}{t \times \frac{V_{Porous\ GaN}}{V_{Bulk\ GaN}}} \qquad (2)$$

Here, $V_{\mathrm{Porous\ GaN}}$ and $V_{\mathrm{Bulk\ GaN}}$ are the filling rates of porous GaN and bulk GaN, respectively. The specific resistance R_{s} and corrected specific resistance R_{correct} were calculated using the sheet resistance R_{sheet} obtained from the Hall measurements as follows:

$$R_s = R_{sheet} \times t \qquad (3),$$

$$R_{coreect} = R_{sheet} \times t \times \frac{V_{Porous\,GaN}}{V_{Bulk\,GaN}} \quad (4)$$

Considering the SEM images and the pore coverage ratio α_p of porous GaN, we assumed that $V_{\text{Porous GaN}}$ and $V_{\text{Bulk GaN}}$ are approximately 50 % and 100 %, respectively.

Figure S3 shows the relationship between the specific resistance and net donor concentration for porous GaN and flat GaN grown by HF-VPE. Porous GaN data were located on the trend line obtained from the flat-GaN data. These results confirm that the free-electron concentration and the specific resistance of porous GaN are determined by the silicon donor concentration and that porous GaN does not contain compensated impurities such as carbon. Thus, the free-electron concentration and the specific resistance of porous GaN can be controlled by the silicon doping concentration in the case of flat GaN.

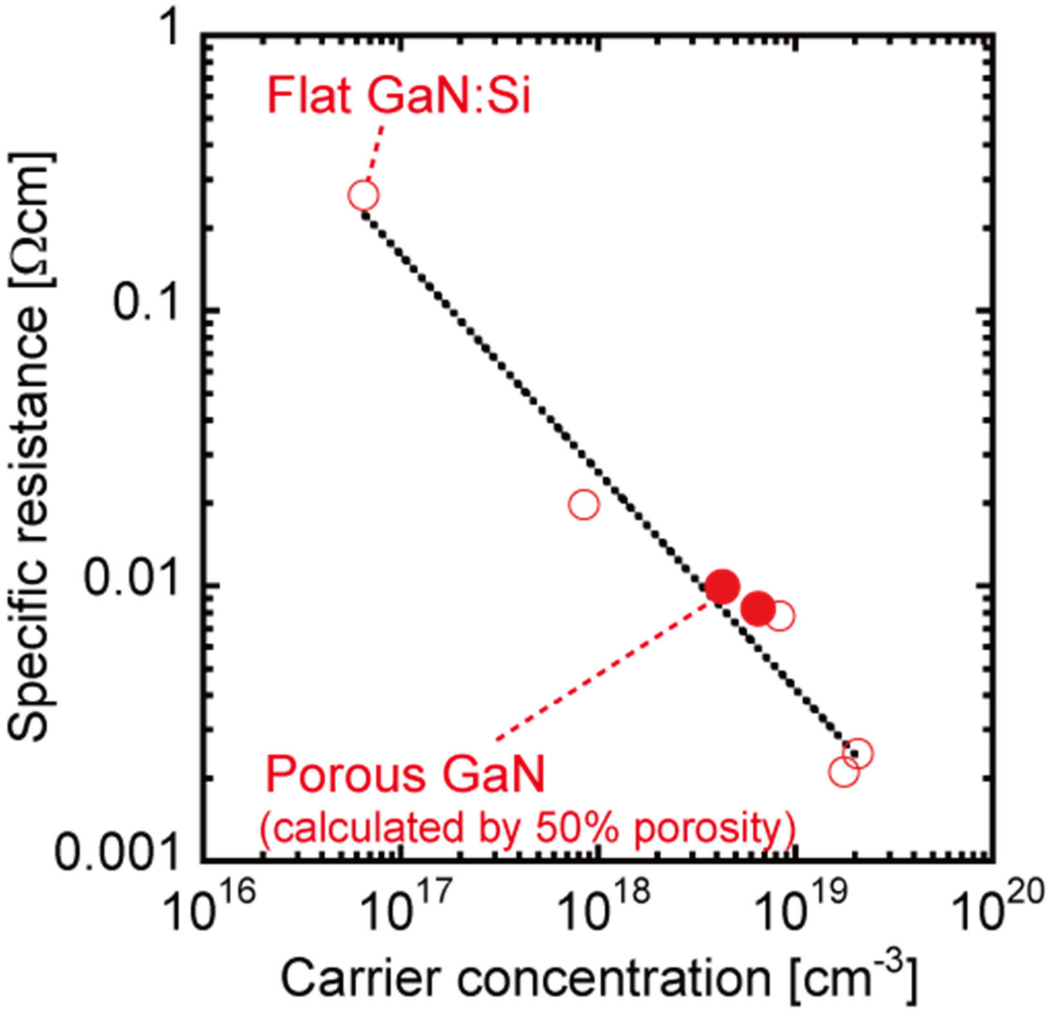

Figure S4. Relationship between the specific resistance and net donor concentration for porous GaN and flat GaN grown by HF-VPE.

7. Mass spectra by LDI

Figure S5(a)–S5(c) show the mass spectra of DHB obtained with each substrate at the maximum point in the map by LDI measurements.

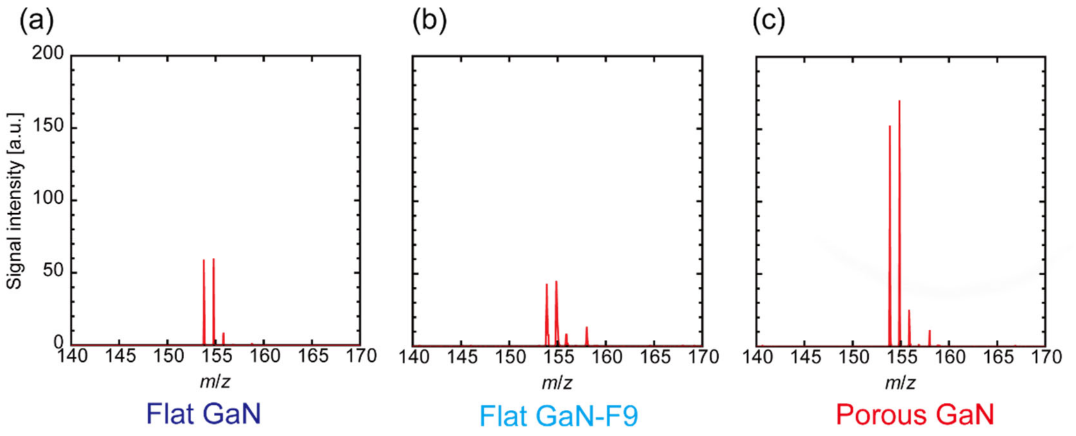

Figure S5. LDI TOF mass spectra of DHB obtained for (a) flat GaN, (b) flat GaN-F9, and (c) porous GaN.

8. Quantum dots photographs and image analysis of those photographs

The substrate contains both porous GaN and flat GaN region, and the edge of this substrate was not formed porous GaN (Figure S6(a)). It can be observed from Figure S6(a) that the attachment of quantum dots are surprisingly different between two region. The image analysis of photograph of Figure 5(e) of manuscript was carried out. Figure S6(b)–S6(d) represent separating Red-Green-Blue (RGB) color information of photograph.

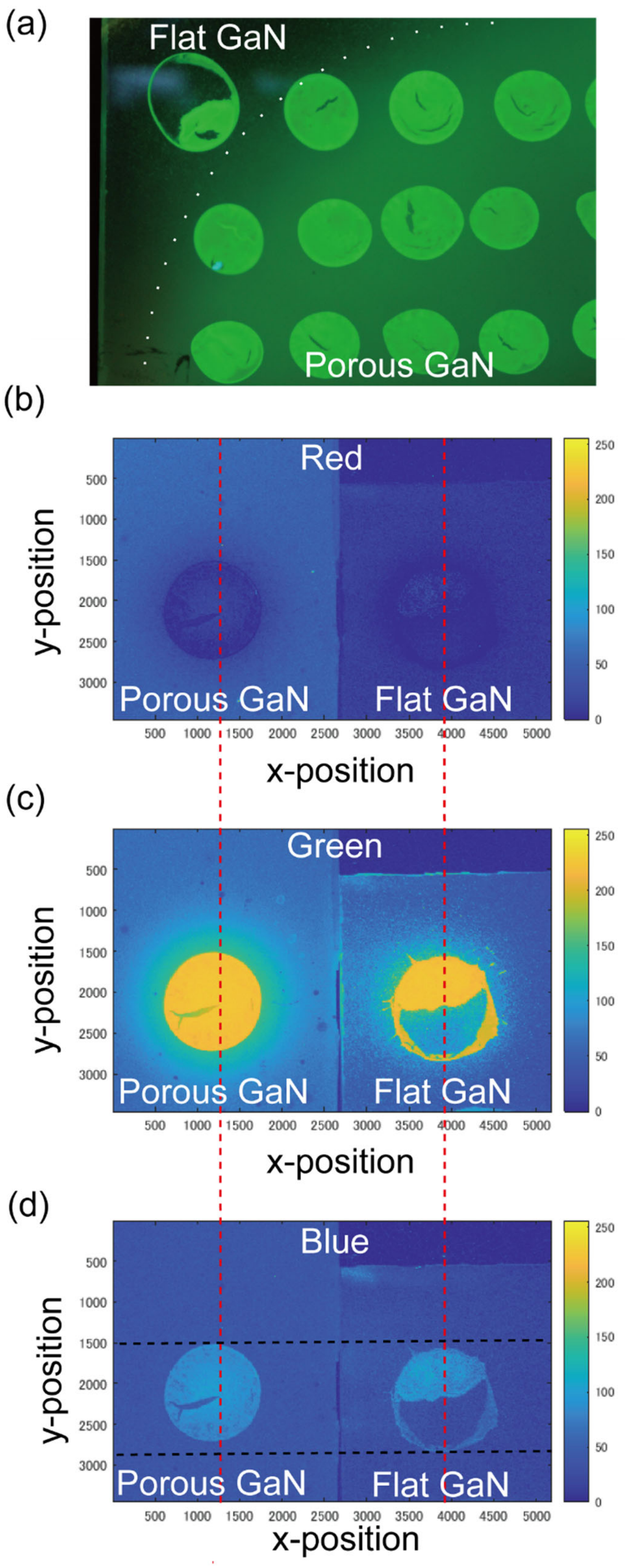
(a)
Flat GaN
Porous GaN
(b)
Red
Porous GaN
Flat GaN
y-position
x-position
(c)
Green
Porous GaN
Flat GaN
y-position
x-position
(d)
Blue
Porous GaN
Flat GaN
y-position
x-position

Figure S6. (a) Photograph if the comparison of attached area CdSe/ZnS core-shell type quantum dots (fluorescence wavelength: 540 nm) attached on substrate under the ultraviolet light. The upper left of the substrate is flat GaN region and lower right of the substrate is porous GaN region. (b)–(d) Separating Red-Green-Blue (RGB) color information of photograph as shown in Figure 5(e) of the manuscript.

9. Photoluminescence spectra of quantum dots on porous GaN and on flat GaN.

Photoluminescence (PL) spectra of quantum dots on porous GaN and on flat GaN upon excitation at λ =350 nm were measured using a FP-6500 spectrometer (JASCO). The samples utilized for PL measurements were those shown in Figure 5(e) in the main manuscript. To normalize the PL intensity form nominally identical surface area of both samples, PL spectra were obtained by utilizing a mask with an aperture of approximately 2 mm-in-diameter. The PL intensity of quantum dots on porous GaN approximately doubled compared to that of quantum dots on flat GaN (shown in Fig. S7). This intensified PL should be achieved by the segregation-free uniform deployment of the quantum dot owing to the petal effect of the porous GaN.

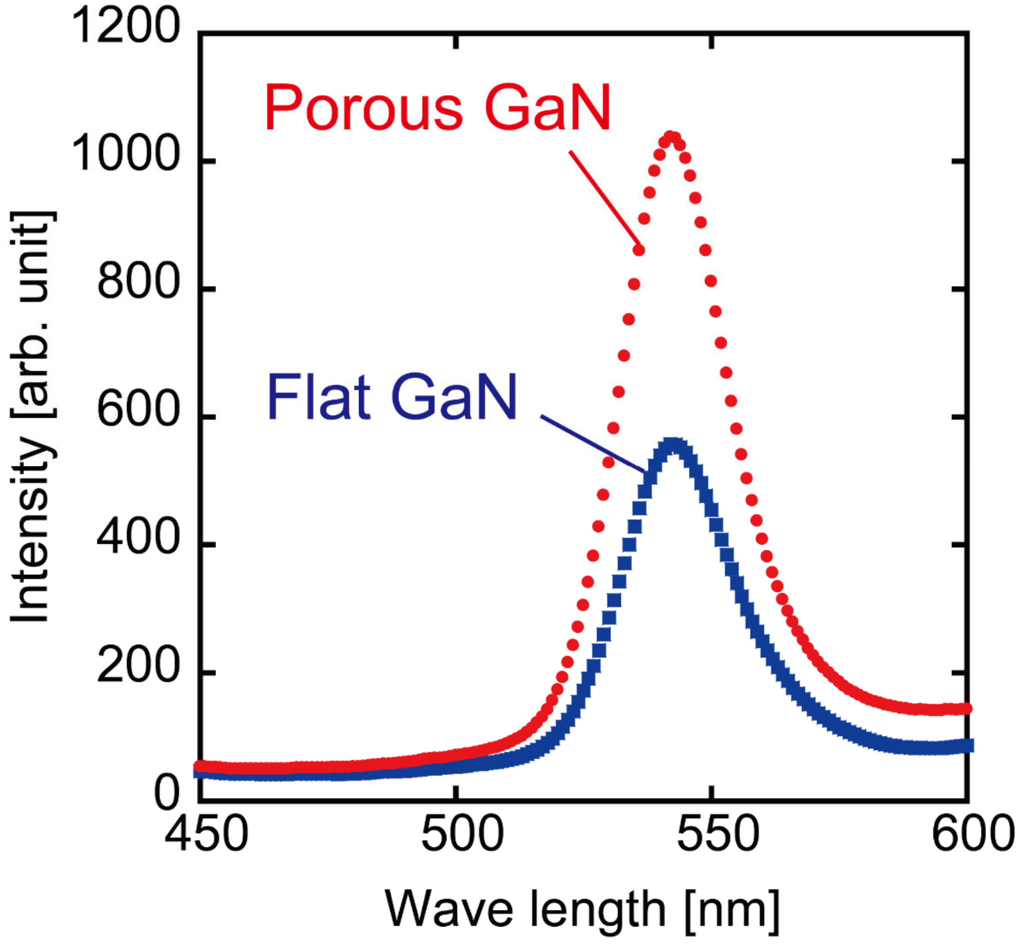

Figure S7. PL spectra of CdSe/ZnS core-shell type quantum dots attached on porous GaN and flat GaN.

10. References

1. Kimura, T.; Aoki, Y.; Horibuchi, K.; Nakamura, D. Nanopipe Formation as a Result of Boron Impurity Segregation in Gallium Nitride Grown by Halogen-Free Vapor Phase Epitaxy. *J. Appl. Phys.* **2016,** 120, 245703.
2. Nakamura, D.; Kimura, T.; Horibuchi, K. Halogen-Free Vapor Phase Epitaxy for High-Rate Growth of GaN Bulk Crystals. *Appl. Phys. Express* **2017,** 10, (4), 045504.
3. Nakamura, D.; Kimura, T. Significant Increase in GaN Growth Rate by Halogen-Free Vapor Phase Epitaxy with Porosity-Controlled Evaporator. *Appl. Phys. Express* **2017,** 10, (9), 095503.
4. Kimura, T.; Horibuchi, K.; Kataoka, K.; Nakamura, D. Macro-Defect-Free Homoepitaxial GaN Growth Through Halogen-Free Vapor-Phase Epitaxy on Native GaN Seeds. *J. Cryst. Growth* **2018,** 494, 17-25.
5. Nakamura, D.; Kimura, T. Ultrahigh-Yield Growth of GaN via Halogen-Free Vapor-Phase Epitaxy. *Appl. Phys. Express* **2018,** 11, (6), 065502.
6. Kimura, T.; Kataoka, K.; Uedono, A.; Amano, H.; Nakamura, D. Growth of high-quality GaN by halogen-free vapor phase epitaxy. *Appl. Phys. Express* **2020,** 13, (8), 085509.
7. Kimura, T.; Sato, S.; Kataoka, K.; Morikawa, T.; Nakamura, D. Self-Assembled Single-Crystalline GaN Having a Bimodal Meso/Macropore Structure To Enhance Photoabsorption and Photocatalytic Reactions. *ACS Applied Materials & Interfaces* **2019,** 11, (4), 4233-4241.
